# *MicroRNA‐206* has a bright application prospect in the diagnosis of cases with oral cancer

**DOI:** 10.1111/jcmm.16598

**Published:** 2021-08-21

**Authors:** Lili Wang, Hongguang Song, Shiming Yang

**Affiliations:** ^1^ Department of Stomatology Chinese PLA General Hospital Beijing China; ^2^ Department of Prosthodontics Tianjin Stomatological Hospital Tianjin China; ^3^ Department of Stomatology Beijing DCN Orthopaedic Hospital Beijing China; ^4^ Otolaryngology Head and Neck Surgery Chinese PLA General Hospital Beijing China

**Keywords:** diagnosis, *MiR‐206*, oral cancer

## Abstract

Previous studies have shown that *microRNA‐206* (*miR‐206*) exhibits anti‐tumour properties in various tumours. Nevertheless, diagnostic significance of *miR‐206* in oral cancer is still poorly known. Our research was carried out to explore the performance of *miR‐206* in the diagnosis of oral cancer. Quantitative real‐time polymerase chain reaction (qRT‐PCR) method was adopted to measure the level of *miR‐206* in serum specimens from oral cancer cases and control individuals. Chi‐square test was performed to analyse the correlation between *miR‐206* level and clinicopathological parameters of the cases. Receiver operating characteristic (ROC) curve was constituted to assess diagnostic accuracy of *miR‐206* in oral cancer. Serum *miR‐206* level in oral cancer patients was significantly lower than that in control individuals (*P* < .001). *miR‐206* expression was obviously related to T classification (*P* = .033), TNM stage (*P* = .008) and lymph node metastasis (*P* = .028). The area under the curve (AUC) of the ROC curve was 0.846 (95% CI = 0.797‐0.896, *P* < .001) with a specificity of 72.7% and a sensitivity of 81.2%. It revealed that *miR‐206* might be a non‐invasive indicator in differentiating oral cancer cases from control individuals. Down‐regulation of *miR‐206* is related to the development of oral cancer. Serum *miR‐206* might be an effective indicator for early detection of oral cancer.

## 1 INTRODUCTION

Oral cancer, a frequent tumour in the world, belongs to head and neck tumours.^1^ In recent years, the morbidity rate of oral cancer is increasing, which seriously affects the quality of the patients' life.^2^ The aetiology of oral cancer is complicated, and relevant aetiological factors of this tumour include smoking, alcohol consumption, the infection of human papillomavirus and genetic and environmental elements.^3^ However, since most cases are diagnosed at advanced stages, overall survival rate of the malignancy is still unsatisfactory.^4^ Surgery, chemotherapy and radiotherapy are main treatments for oral cancer, but there are some limitations in these methods.^5^ Thus, searching for novel diagnostic factors and therapeutic targets is an urgent need for oral cancer.

MicroRNAs (MiRNAs) are a group of short and conservative non‐coding RNAs, which play important roles in multiple biological process.^6^ Accumulated evidence supported that the dysregulation of miRNAs was related to the pathogenesis and progression of human cancers.^7^ *MicroRNA‐206* (*miR‐206*), seating on human chromosome 6p12.2, was considered to be a tissue‐specific miRNA associated with the differentiation of skeletal muscle.^8^ More and more proofs unveiled that *miR‐206* acted as an anti‐oncogene and its decreased level was detected in many human tumours.^9^, ^10^ Previous studies suggested that high levels of *miR‐206* could suppress tumour cell growth, migration and invasion, and induced their apoptosis.^11^, ^12^ Besides, *miR‐206* was proven to hold certain significance in the diagnosis, treatment and prognosis of tumours.^13^, ^14^ Nevertheless, diagnostic ability of *miR‐206* in oral cancer has not been studied.

In our research, we detected the level of *miR‐206* in serum among oral cancer cases and analysed the correlation between *miR‐206* level and clinicopathological parameters of the cases. The potential of serum *miR‐206* as a diagnostic indicator for oral cancer has also been studied.

## 2 MATERIALS AND METHODS

### 2.1 Participants and specimens

Our research was authorized by the Ethical Committee of Chinese PLA General Hospital (No. JSH2016OSCC002). All patients for our study signed written informed consent. We enrolled 132 oral cancer patients who were histopathologically diagnosed by experienced pathologists at Chinese PLA General Hospital. Patients were more than 18 years older and had no history of other cancers or oral diseases. Before our research, cases had not received any radio‐ or chemotherapy treatments. Besides, 85 healthy people served as controls.

5 mL peripheral blood was obtained from each participant after overnight fasting. Then, serum was separated from blood sample through centrifugation at 1500 *g* for 10 min. Supernate was at once kept at −80℃ until use. Clinical data of the cases are listed in Table 1, including age, gender, T classification, histological grade, TNM stage and lymph node metastasis.

**TABLE 1:** Relationship between *miR‐206* expression and clinical features of oral cancer patients

Clinical features	Cases (n = 132)	*miR‐206* expression		*χ* ^2^	*P*
		High (n = 59)	Low (n = 73)		
Age (years)					
≤57	70	32	38	0.062	.803
>57	62	27	35		
Gender					
Male	87	36	51	1.136	.286
Female	45	23	22		
Smoking					
No	51	26	25	1.327	.249
Yes	81	33	48		
Alcohol consumption					
No	67	34	33	2.014	.156
Yes	65	25	40		
T classification					
T1‐T2	76	40	36	4.563	.033
T3‐T4	56	19	37		
Histological grade					
Well/moderate	79	37	42	0.364	.546
Poor	53	22	31		
TNM stage					
Ⅰ‐Ⅱ	68	38	30	7.099	.008
Ⅲ‐Ⅳ	64	21	43		
Lymph node metastasis					
Negative	85	44	41	4.824	.028
Positive	47	15	32		

### 2.2 QRT‐PCR

According to the protocol of the manufacturer, miRNeasy mini kit (Qiagen) was employed to isolate miRNAs from serum samples. The quality and quantity of RNA were detected via NanoDrop 2000 Spectrophotometer (NanoDrop Technologies). TaqMan MicroRNA Reverse Transcription Kit (Applied Biosystems) was adopted to perform reverse transcription. Primers for reverse transcription contained 5′‐CTCAGCGGCTGTCGTGGACTGCGCGCTGCCGCTGAGCCACACAC‐3′ for *miR‐206* and 5′‐CTCGCTTCGGCAGCACA‐3′ for *U6*. qRT‐PCR was conducted with 40 cycles of denaturing at 95°C for 15 s, annealing at 60°C for 20 s and extending at 72°C for 20 s, utilizing SYBR Real‐Time PCR kit (GenePharma) with 7500 Real‐Time PCR system (Applied Biosystems) based on instruction book. *U6* acted as endogenous reference. Primer sequences were as follows: *miR‐206*, sense‐5′‐CGTCAGAAGGAATGATGCACAG‐3′ and anti‐sense‐5′‐ACCTGCGTAGGTAGTTTCATGT‐3′; and *U6*, sense‐5′‐CTCGCTTCGGCAGCACA‐3′ and anti‐sense‐5′‐AACGCTTCACGAATTTGCGT‐3′. Relative quantification of *miR‐206* expression was analysed via 2^−ΔΔCt^ method. All data analysed were from three independent experiments repeated in triplicate.

### 2.3 Statistical analysis

We used SPSS 18.0 software and GraphPad Prism 5.0 software to analyse all data. Data were presented as mean ± standard deviation (SD). Different levels of *miR‐206* between oral cancer specimens and matched control specimens were compared with Student's *t* test. The correlation between *miR‐206* level and clinicopathological parameters of the cases was assessed by chi‐square test. Receiver operating characteristic (ROC) curve was constructed to assess diagnostic ability of *miR‐206* in oral cancer. *P* < .05 was seen as significant threshold.

## 3 RESULTS

### 3.1 Basic characteristics of enrolled participants

Clinical characteristics of oral cancer patients are shown in Table 1. 45 women and 87 men with a mean age of 57.39 ± 19.28 years (range, 41‐78 years) were enrolled in our research. There were 81 smokers and 65 drinkers. 76 patients had T classification of T1‐T2 and 56 at T3‐T4. Histological grade was well/moderate in 79 patients and poor in the remaining 53 ones. According to TNM staging system, 68 patients were at stage Ⅰ‐Ⅱ and 64 at stage Ⅲ‐Ⅳ. Moreover, among these patients, 47 had positive lymph node metastasis.

### 3.2 Serum *miR‐206* level was reduced in oral cancer cases

**FIGURE 1:**
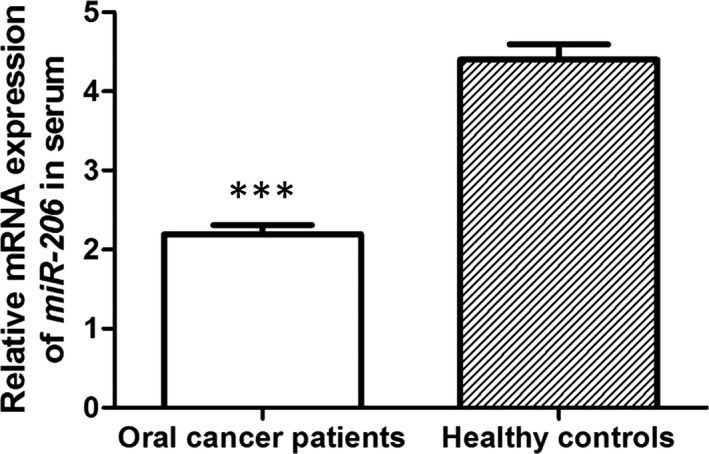
Relative expression levels of *miR‐206* mRNA in oral cancer patients and healthy individuals. Compared with healthy controls, serum *miR‐206* level was significantly down‐regulated in oral cancer patients at mRNA level. *** indicated *P* < .001

Quantitative real‐time polymerase chain reaction was adopted to measure the level of serum *miR‐206* in oral cancer cases and control volunteers. The results revealed that the expression of serum *miR‐206* in oral cancer cases was significantly lower than that in control volunteers (*P* < .001; Figure 1).

### 3.3 Association between *miR‐206* level and clinicopathological parameters of oral cancer cases

Using the mean serum *miR‐206* level as a cut‐off value, 132 oral cancer cases were classified into high *miR‐206* level group (n = 59) and low *miR‐206* level group (n = 73). The correlation between *miR‐206* level and clinicopathological parameters of cancer cases was analysed via chi‐square test. The results indicated that *miR‐206* level was obviously related to T classification (*P* = .033), TNM stage (*P* = .008) and lymph node metastasis (*P* = .028) (Table 1). Nevertheless, no significant correlation was found between *miR‐206* level and age, gender, smoking, alcohol consumption or histological grade (all, *P* > .05; Table 1).

### 3.4 Diagnostic ability of *miR‐206* in oral cancer cases

**FIGURE 2:**
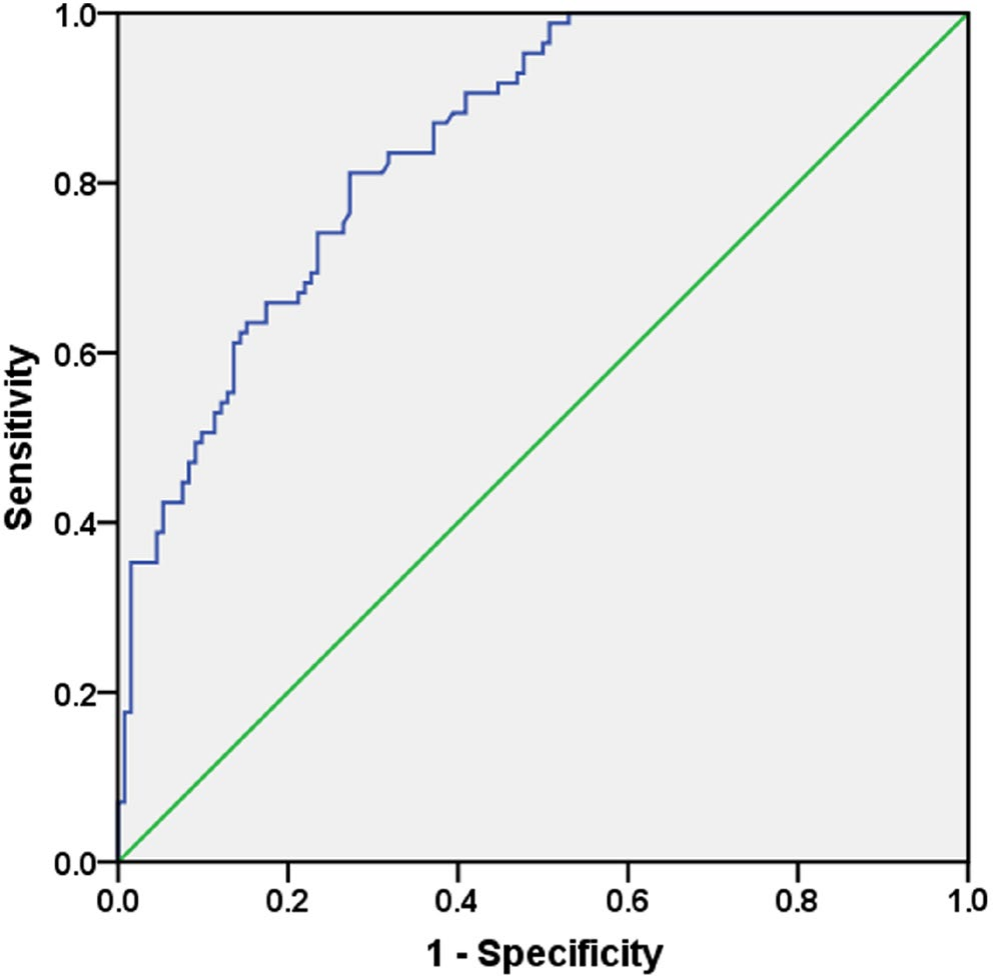
ROC curve analysis of serum *miR‐206* in the diagnosis of oral cancer patients. The AUC was 0.846 with a sensitivity of 81.2% and a specificity of 72.7%

Receiver operating characteristic curve analysis was conducted to investigate whether *miR‐206* could be adopted in the diagnosis of oral cancer. As shown in Figure 2, the area under the curve (AUC) was 0.846 (95% CI = 0.797‐0.896, *P* < .001) with a sensitivity of 81.2% and a specificity of 72.7%. The optimal cut‐off value for serum *miR‐206* level to diagnose the disease was 2.88. Serum *miR‐206* might be a valuable indicator for the diagnosis of oral cancer cases.

## 4 DISCUSSION

Accumulated documents reported that miRNAs were related to carcinogenesis and the progression of several tumours via acting as cancer genes or anti‐oncogenes.^15^ Nevertheless, their functions in oral cancer are not fully understood. In our research, we proved that serum *miR‐206* level was significantly reduced in oral cancer patients compared to healthy controls. We also assessed the correlation between *miR‐206* level and clinicopathological parameters of the cases. As a result, patients with low level of *miR‐206* more frequently faced advanced T classification, advanced TNM stage and positive lymph node metastasis, all of which were aggressive clinicopathological parameters representing advanced development and metastasis of tumours. In ROC curve analysis, serum *miR‐206* functioned as a forceful indicator to distinguish oral cancer cases from control people with satisfactory AUC, sensitivity and specificity.

*miR‐206* belongs to miR‐1 family which includes muscle‐specific miRNAs and is involved in the development of muscle.^16^ More and more proofs indicated that *miR‐206* was abnormally expressed in many cancers. For example, Chen et al reported that the level of *miR‐206* was reduced in lung adenocarcinoma tissue samples and cells and that its up‐regulation could suppress cell viability, migration and angiogenesis.^17^ Liang et al suggested that *miR‐206* was down‐regulated in triple‐negative breast cancer tissue samples and cells. Heightened level of *miR‐206* might be involved in the invasion and angiogenesis of breast cancer.^18^ Moreover, Bao et al proved that decreased level of *miR‐206* was closely related to advanced clinical stage and T classification and positive metastasis in osteosarcoma patients.^19^ According to Yunqiao et al, low *miR‐206* level occurred more often in hepatocellular carcinoma patients with positive lymph node metastasis and advanced TNM stage.^12^ Similar to above‐mentioned achievements, our findings revealed that *miR‐206* could serve as an anti‐oncogene in oral cancer and participate in the disease pathogenesis and development. However, serum *miR‐206* level was not related to histological grade of oral cancer patients in our research, which was inconsistent with previous findings.^20^, ^21^ The role of *miR‐206* in different cancers might be varied. Besides, sample size and source might cause differences as well. Considering these limitations, further studies are required.

Accumulated data suggested that *miR‐206* was related to overall survival, disease‐free survival and prognosis of many cancers, such as oral squamous cell carcinoma.^22^, ^23^, ^24^ Nevertheless, diagnostic ability of *miR‐206* in oral cancer has not been studied. MiRNAs are stable and easily detected in some body fluids, such as blood, serum and plasma, suggesting that they could become non‐invasive indicators for the diagnosis and prognosis of tumours.^25^ A research scheduled by Tan et al mentioned that circulating *miR‐206* could be a potential diagnostic factor for hepatocellular carcinoma.^26^ Such result was consistent with ours that diagnostic ability of serum *miR‐206* was strong in oral cancer.

In oestrogen receptor–positive breast cancer, TGF‐β signalling pathway was a target of *miR‐206* in inhibiting epithelial mesenchymal transition.^27^ Cai et al proved that *miR‐206* inhibited the growth, migration and invasion of renal cell carcinoma cells via targeting VEGF‐A directly.^28^ Based to earlier researches, we hypothesized that *miR‐206* participated in tumorigenesis and disease progression via regulating diverse genes in different processes of human tumours. Nevertheless, exact molecular mechanism of *miR‐206* functioning in oral cancer remains unclear.

In conclusion, serum *miR‐206* level is reduced in oral cancer patients and related to the development of this tumour. Moreover, serum *miR‐206* could be an effective bio‐marker for early diagnosis of oral cancer.

## CONFLICT OF INTEREST

None.

## AUTHOR CONTRIBUTIONS

L.W. conceived and designed the experiments; L.W., H.S. conceived and performed the experiments; S.Y. prepared figures. H.S., S.Y. wrote the main manuscript text. All authors reviewed the manuscript.

## Data Availability

All data generated or analysed in our research are included in this article.
